# Gcap14 is a microtubule plus-end-tracking protein coordinating microtubule–actin crosstalk during neurodevelopment

**DOI:** 10.1073/pnas.2214507120

**Published:** 2023-02-16

**Authors:** Dong Jin Mun, Bon Seong Goo, Bo Kyoung Suh, Ji-Ho Hong, Youngsik Woo, Soo Jeong Kim, Seunghyun Kim, Su Been Lee, Yubin Won, Jin Yeong Yoo, Eunbyul Cho, Eun Jin Jang, Truong Thi My Nhung, Hong Minh Triet, Hongyul An, Haeryun Lee, Minh Dang Nguyen, Seung-Yeol Park, Seung Tae Baek, Sang Ki Park

**Affiliations:** ^a^Department of Life Sciences, Pohang University of Science and Technology, Pohang 37673, Republic of Korea; ^b^Department of Pediatrics, Seoul National University College of Medicine, Seoul National University Children's Hospital, Seoul 03080, Korea; ^c^Department of Clinical Neurosciences, Hotchkiss Brain Institute, Alberta Children’s Hospital Research Institute, University of Calgary, Calgary, AB T2N 4N1, Canada; ^d^Department of Cell Biology and Anatomy, Hotchkiss Brain Institute, Alberta Children’s Hospital Research Institute, University of Calgary, Calgary, AB T2N 4N1, Canada; ^e^Department of Biochemistry and Molecular Biology, Hotchkiss Brain Institute, Alberta Children’s Hospital Research Institute, University of Calgary, Calgary, AB T2N 4N1, Canada

**Keywords:** neurodevelopment, neuronal migration, microtubule plus-end-tracking protein, microtubule–actin crosstalk, microtubule dynamics

## Abstract

Regulation of microtubule dynamics is required to properly control various steps of neurodevelopment. In this study, we identified granule cell antiserum-positive 14 (Gcap14) as a microtubule plus-end-tracking protein and as a regulator of microtubule dynamics during neurodevelopment. *Gcap14* knockout mice exhibited impaired cortical lamination. Gcap14 deficiency resulted in defective neuronal migration. Moreover, nuclear distribution element nudE-like 1 (Ndel1), an interacting partner of Gcap14, effectively corrected the downregulation of microtubule dynamics and the defects in neuronal migration caused by Gcap14 deficiency. Finally, we found that the Gcap14–Ndel1 complex participates in the functional link between microtubule and actin filament, thereby regulating their crosstalks in the growth cones of cortical neurons. Taken together, we propose that the Gcap14–Ndel1 complex is fundamental for cytoskeletal remodeling during neurodevelopmental processes such as neuronal processes elongation and neuronal migration.

## Significance

Tight regulation of cytoskeletal remodeling is essential for various neurodevelopmental processes. The microtubule and actin crosstalk to accomplish dynamic rearrangement of the cytoskeleton. Here, we reveal that granule cell antiserum-positive 14 (Gcap14) functions as a microtubule plus-end-tracking protein modulating microtubule dynamics. These processes are achieved by Gcap14 in complex with Ndel1, which functionally links the microtubule and actin cytoskeleton. Successful linkage of growing microtubules to actin filament requires the Gcap14–Ndel1 complex in the growth cones of developing neurons, underlying the proper neuronal migration and cortical lamination. These findings provide fundamental mechanistic insights into the cytoskeletal dynamics regulation required for key neurodevelopment steps.

Cortical development requires tightly regulated control over the timing of neurogenesis, neuronal migration, and maturation to acquire the lamination of stratified cortical layers (CLs) (1). During this process, the microtubules undergo a cycle of rapid growth and disassembly, known as dynamic instability (2). The microtubules dynamically reorganize to differentiate spatially and temporally depending on functional contexts and generate pushing and pulling forces (3, 4). These forces allow dynamic neuronal morphogenesis processes, such as the formation of the leading process of migrating cells and the elongation of neurites.

Microtubule dynamics are regulated by microtubule-associated proteins (MAPs), including microtubule plus-end-tracking proteins (+TIPs). The +TIPs accumulate at the plus ends of growing microtubules (5–7) and play important roles in neurodevelopmental processes, including neurite outgrowth, axon branching, and synaptic maturation. For example, cytoplasmic linker protein 170 (CLIP170), cytoskeleton-associated protein 5-A (XMAP215), and transforming acidic coiled-coil (CC)–containing protein 3 (TACC3) promote axon formation and regulate axon outgrowth (8–10). CLIP-associating protein (CLASP) and adenomatous polyposis coli (APC) function as modulators of growth cone steering (11–14). Recently, +TIPs have also been studied as cross-linking proteins for microtubule–actin crosstalk. In the crosstalk between microtubules and actin, +TIPs provide direct physical bridges or indirectly contribute through shared regulators to the dynamic behavior of microtubules and actin (15, 16). Microtubule–actin crosstalk is important for core biological processes, including cell division, migration, and morphogenesis (17–19). Although the link between +TIPs and microtubule–actin crosstalk in the nervous system has been implicated in neurodevelopmental processes such as neuronal migration and differentiation, the neuron-specific mechanistic basis has not been clearly elucidated.

Nuclear distribution element nudE-like 1 (NDEL1) is a regulator of neuronal development (20). Deficiency in NDEL1 results in impaired neuronal precursor proliferation and neuronal migration (21, 22). In addition, human genetic association studies on NDEL1 have provided evidence for its involvement in several neurodevelopmental disorders, such as schizophrenia and microcephaly (21, 23–26). NDEL1 regulates the dynamics of microtubules and actin (27–29). In association with the dynein motor and LIS1, NDEL1 stabilizes the microtubule and affects molecular trafficking along microtubules (28, 30). Moreover, NDEL1 cooperates with the actin-binding protein TRIO-associated repeat on actin (TARA) to control filamentous actin dynamics (27, 31). These cytoskeletal functions are known to regulate neuronal development, but the corresponding factors that link microtubules and actin are unknown.

Granule cell antiserum-positive 14 (Gcap14, also known as CCSER2, FAM190B, KIAA1128, NPD012) is a CC domain–containing protein with multiple isoforms generated by alternative splicing that functions as an MAP. Additionally, according to the Biological General Repository for Interaction Datasets and STRING (32, 33), the majority of Gcap14 interacting partners are functionally related to brain development and regulation of the microtubule cytoskeleton. Gcap14 has been reported to be highly expressed in the brain and binds to microtubules (34), but its neural functions remain unknown. Here, we identified that Gcap14 is a +TIP that functions as a regulator of microtubule dynamics, thereby modulating neocortical development. Our results also reveal that the neurodevelopmental functions of Gcap14 are achieved in collaboration with Ndel1 by bridging the microtubules and actin cytoskeletons.

## Results

### Gcap14 knockout (KO) Mouse Shows Impaired Cortical Lamination.

**Fig. 1.**
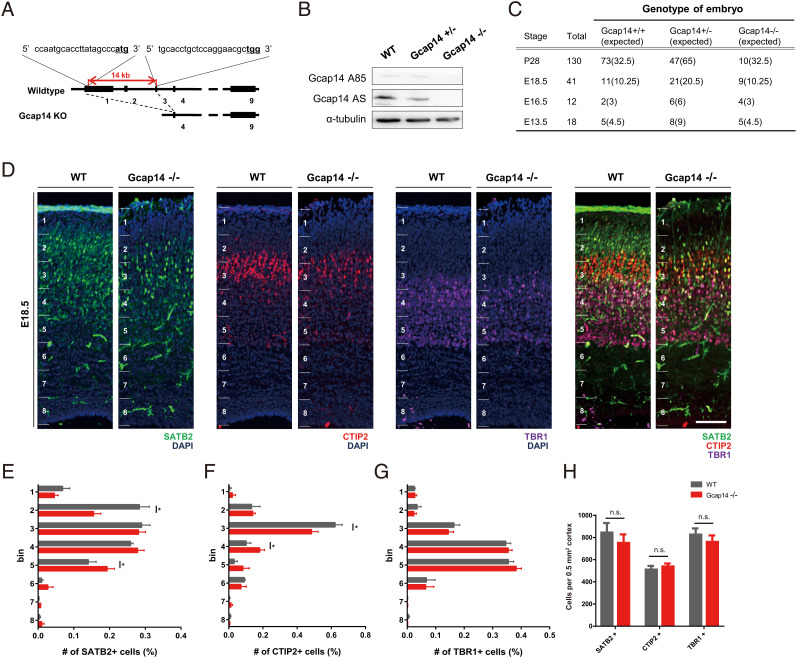
*Gcap14* KO mice show impaired cortical development. (*A*) Schematic diagram of the *Gcap14* targeting strategy. The gRNA-targeting sequences in exons 1 and 3 are shown, and the protospacer adjacent motif (PAM) sequences are underlined. (*B*) Western blots against Gcap14 in E18.5 brain lysate. (*C*) Table showing the number of embryos at the stages that were collected from female partners of *Gcap14*^+/−^ intercrosses and genotyped. (*D*) Representative image for immunostaining of layer markers in E18.5 cortical sections. (The scale bar represents 100 μm.) (*E*–*G*) Quantification of the SATB2 positive (*E*), CTIP2 positive, (*F*), and TBR1 positive cell fraction *(G)* (Student’s *t* test, SATB2 positive cell fraction: significant decrease in bin 2, *P = 0.0113, and significant increase in bin 5, *P = 0.0302; CTIP2 positive cell fraction: significant decrease in bin 3, **P = 0.0026, and significant increase in bin 4, **P = 0.0063; TBR1 positive cell fraction: all not significant). (*H*) Quantification of cell number per 0.5 mm^2^ cortex. All results are presented as mean ± SEM (control, n = 4 brains; Gcap14 shRNA, n = 4 brains). No significant changes were observed between WT and Gcap14 −/− (Student’s *t* test, all not significant, *P* > 0.05). Data information: Error bars are reported as ± SEM. *P < 0.05, **P < 0.01, ***P < 0.001; n.s., not significant by Student’s *t* test.

To investigate the role of Gcap14 in brain development, we generated a *Gcap14* KO mouse using CRISPR/Cas9-mediated genome editing. The *Gcap14* KO mouse contains a 14kb deletion from exon1 to exon3 (Fig. 1*A*). We confirmed the depletion of Gcap14 protein levels in the whole brain of *Gcap14^−/−^* mice (Fig. 1*B*). *Gcap14^+/−^* intercrosses yielded fewer *Gcap14^−/−^* mice than expected (Fig. 1*C*), demonstrating perinatal lethality without abnormalities in body or brain weight (*SI Appendix*, Fig. S1 *A*–*C*). Histological characterization using hematoxylin and eosin staining revealed a slightly thickened cortex in *Gcap14* KO mice (*SI Appendix*, Fig. S1 *D*–*G*).

As the *Gcap14* mRNA level peaked at the late embryonic stage (*SI Appendix*, Fig. S2 *A* and *B*), we then analyzed the effects of *Gcap14* deficiency on cortical development. To assess CL organization that requires proper neuronal migration, we performed immunohistochemical analyses of E18.5 cortices using layer markers such as SATB2, CTIP2, and TBR1 (Fig. 1*D*). Cortical areas were subdivided into eight parts (1 to 8), and layer marker-positive cells in each area were analyzed (Fig. 1 *E*–*G*). While SATB2-positive neurons were predominantly distributed through areas 2 to 4 in the wild type, those neurons were more enriched in areas 2 to 5 of the *Gcap14^−/−^* cortex. CTIP2-positive neurons were predominantly observed in area 3 in the wild type, while they were broadly distributed in areas 3 and 4 in the *Gcap14^−/−^* cortex. The distribution of TBR1-positive neurons in the wild-type and *Gcap14*-deficient cortices was not distinguishable. Layer IV pyramidal (SATB2 positive) and subcerebral (CTIP2 positive) neurons that were produced after E13.5 were critically affected by Gcap14 deficiency, compared to corticothalamic (TBR1 positive) neurons. Additionally, the total cell numbers in KO mice in the corresponding cortical area were not altered (Fig. 1*H*). These data indicate that *Gcap14*-deficient mice exhibit impaired cortical lamination.

### Gcap14 Regulates Neuronal Migration in the Developing Neocortex.

**Fig. 2.**
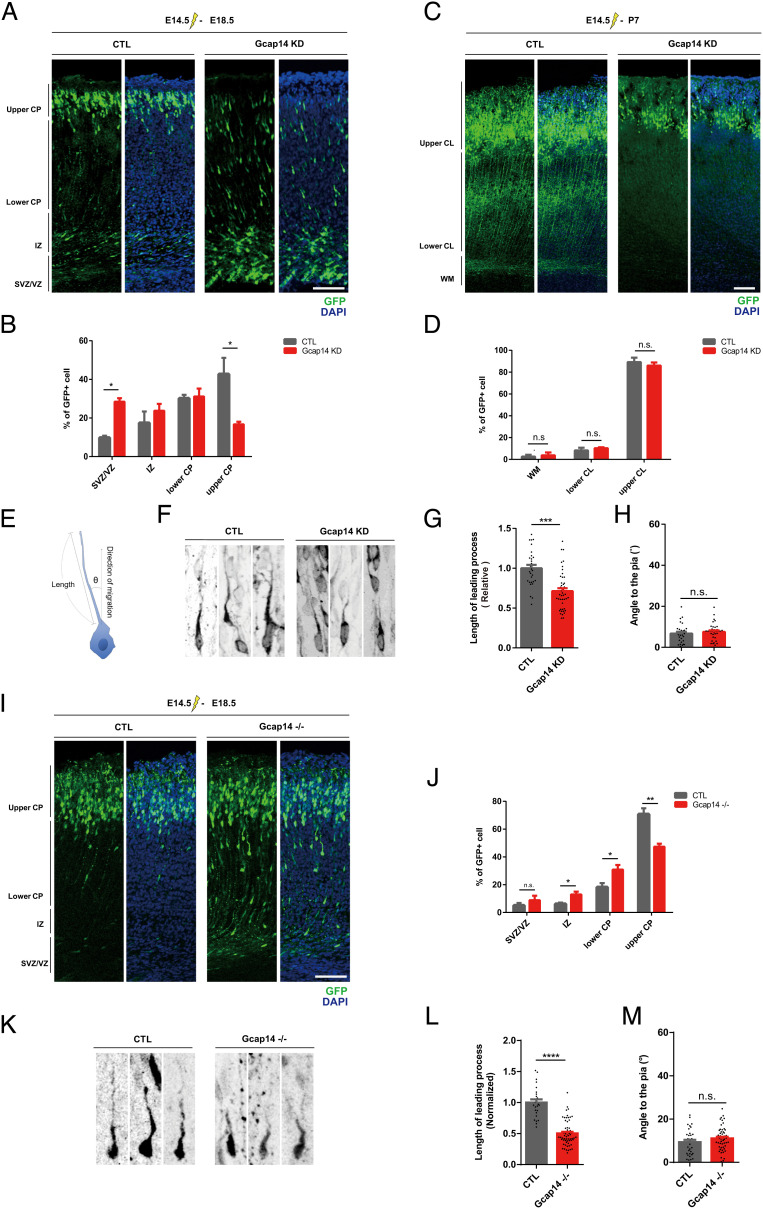
Gcap14 regulates neurodevelopmental processes, including neuronal migration. (*A* and *C*) Representative image of the brain of mouse embryos that were electroporated in utero with shRNA plasmids at E14.5 and analyzed at E18.5 (*A*) and P7 (*C*). Coronal sections of the cortex illustrating transfected GFP-positive neurons are shown (control, CTL, n = 3 brains; Gcap14 KD, n = 3 brains). (The scale bars represent 100 μm.) (*B* and *D*) Quantitative analyses of the transfected cell distribution of the brain at E18.5 (*B*) (Student’s *t* test, SVZ/VZ: significant increase, **P = 0.0022; upper CP: significant decrease, *P = 0.0416) and P7 (*D*) (Student’s *t* test, all not significant, *P* > 0.05). (*E*) Schematic diagram of the length and orientation of leading processes for analyses. The angle was measured by using the closest connecting line from the cell soma to the pia. (*F*) Morphological analyses of the leading processes of migrating neurons (control, n = 29 cells; Gcap14 shRNA, n = 35 cells). (*G* and *H*) Quantitative analysis of the length (*G*) (Student’s *t* test, ****P < 0.0001) and angle (*H*) (Student’s *t* test, all not significant, *P* > 0.05) of leading processes. (*I*) Representative image of the brain of mouse embryos that were electroporated in utero with GFP-expressing plasmids at E14.5 and analyzed at E18.5. Coronal sections of the cortex illustrating transfected GFP-positive neurons are shown (control, CTL, n = 3 brains; Gcap14 −/−, n = 3 brains). (The scale bar represents 100 μm.) (*J*) Quantitative analyses of the transfected cell distribution of the brain at E18.5 (*I*) (Student’s *t* test, IZ: significant increase, *P = 0.0391; lower CP: significant increase, *P = 0.0477; upper CP: significant decrease, **P = 0.0084). (*K*) Morphological analyses of the leading processes of migrating neurons (CTL, n = 25 cells; Gcap14 −/−, n = 62 cells). (*L* and *M*) Quantitative analysis of the length (*L*) (Student’s *t* test, ****P < 0.0001) and angle (*M*) (Student’s *t* test, all not significant, *P* > 0.05) of leading processes. Data information: Error bars are reported as ± SEM. *P < 0.05, **P < 0.01, ***P < 0.001; n.s., not significant by Student’s *t* test.

To further investigate the role of Gcap14 during neuronal migration, we performed in utero electroporation (IUE) using a Gcap14 small hairpin RNA (shRNA) construct at embryonic day 14.5 (E14.5) and analyzed the distribution of transfected GFP-positive cells within the cortex at E18.5 and postnatal day 7 (P7). At E18.5, while GFP (+) neurons in control brains migrated into the upper cortical plate (CP), Gcap14 knockdown (KD) neurons remained in the subventricular zone (SVZ) or ventricular zone (VZ) with fewer neurons in the upper CP (Fig. 2 *A* and *B* and *SI Appendix*, Fig. S3 *A*–*D*). Additionally, Gcap14-deficient neurons in the upper CP exhibited a slight increase in soma size (*SI Appendix*, Fig. S4 *A* and *B*). On the contrary, at P7, both the control GFP (+) neurons and Gcap14 KD neurons migrate into the upper CL (Fig. 2 *C* and *D*). In sum, these data indicate that Gcap14 regulates the molecular processes for cortical neuronal migration.

Migrating neurons are highly polarized to the direction of movement, which is achieved by directional remodeling of the leading process (35). Therefore, we examined the length and orientation of the leading process of neurons migrating through the lower CP (Fig. 2*E*). In agreement with the migration delay, Gcap14 KD caused a decrease in the length without changing the orientation of the leading process (Fig. 2 *F*–*H*). These data suggest that Gcap14 affects the leading process in the cytoskeletal remodeling of migrating neurons.

In addition, we analyzed migrating neurons in the developing *Gcap14* KO brain using IUE with the GFP-expression construct. While the transfected cells in WT brains migrated into the upper CP, cells in the Gcap14 KO brain neurons showed a delay in neuronal migration (Fig. 2 *I* and *J*). The migrating cells in the Gcap14 KO brain also showed a decrease in the length without changing the orientation of the leading process (Fig. 2 *K*–*M*). The phenotypic severity of the KO condition was lower than that of the KD condition, likely due to potential compensatory mechanisms. Also, we could not entirely rule out the possibility of off-target effects of the Gcap14 shRNA by rigorous rescue experiments because Gcap14 overexpression itself caused significant migration defects. Nevertheless, the phenotypic similarity of KD and KO further supports the roles of Gcap14 in the neuronal migration process.

### Gcap14 Localizes at the Microtubule Plus End and Regulates Microtubule Dynamics.

**Fig. 3.**
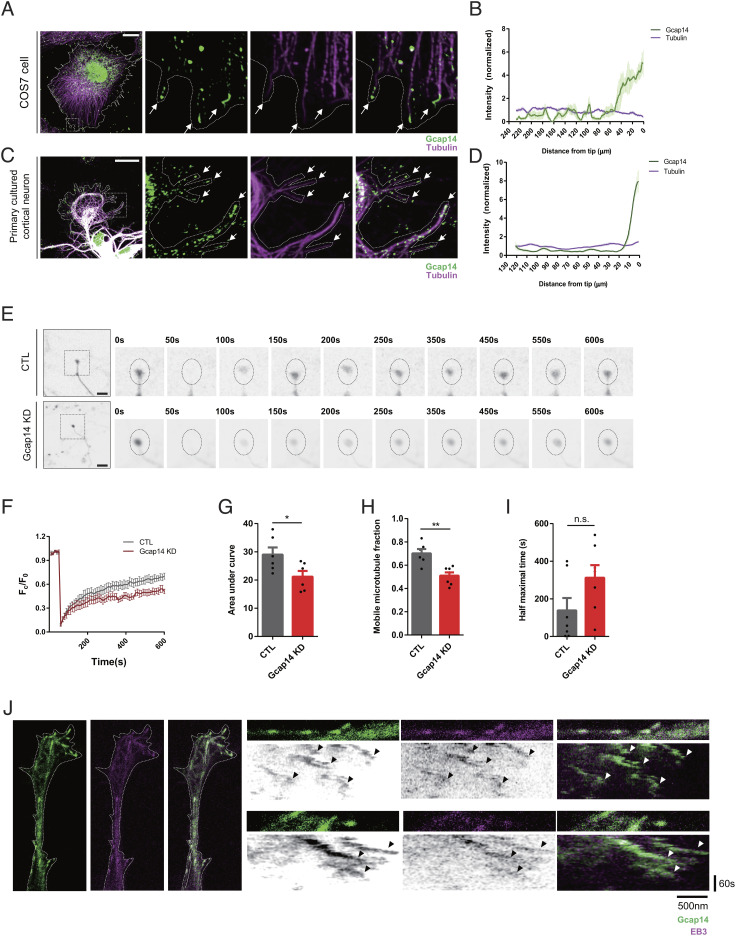
Gcap14 localizes at microtubule plus ends and controls microtubule dynamics. (*A* and *C*) Immunocytochemical analyses of endogenous Gcap14 localization in the COS7 cell and the primary cultured mouse cortical neuron. Each scale bar represents 10 μm (*A*) and 20 μm (*C*). (*B* and *D*) Quantification of the anti-Gcap14 and anti-α-tubulin signals along the microtubule is shown. The arrows indicate microtubule plus ends with Gcap14 signals. (*E*) Representative time-lapse images of the FRAP assay on the axon tips of cortical neurons expressing mCherry-α-tubulin. The dashed circle indicates the region of photobleaching (10% 568 nm laser for 10 s). (The scale bar represents 10 μm.) (n > 6). (*F*–*I*) Quantification of fluorescence recovery over time after photobleaching (*F*). Analyses of areas under curves (*G*) (Student’s *t* test, *P = 0.038), the fraction of mobile microtubule (*H*) (Student’s *t* test, **P = 0.0025), and average half-maximal time of fluorescence recovery (*I*) (Student’s *t* test, all not significant, *P* > 0.05). (*J*) Representative live cell images of the GFP–Gcap14 and RFP–EB3 dynamics on the axon of cortical neurons and kymograph of Gcap14 and EB3 movements. Data information: Error bars are reported as ± SEM. *P < 0.05, **P < 0.01, ***P < 0.001; n.s., not significant by Student’s *t* test.

To investigate the neuronal function of Gcap14, we performed immunocytochemical analyses in COS7 cells (Fig. 3 *A* and *B*) and cultured primary cortical neurons (Fig. 3 *C* and *D*). Interestingly, the Gcap14 signal was significantly enriched at the microtubule tips, showing more significant enrichment at the plus end (Fig. 3 *A*–*D*). Ectopically expressed Gcap14 isoforms, AS and A85, also exhibited an affinity for the microtubule plus end (*SI Appendix*, Fig. S5 *A* and *B*). Additionally, in the microtubule cosedimentation assay, Gcap14 was enriched in the microtubule fraction (*SI Appendix*, Fig. S5 *C* and *D*). These results indicate that Gcap14 is a +TIP.

To determine whether Gcap14 regulates microtubule dynamics as a +TIP, we performed the fluorescence recovery after photobleaching (FRAP) assay in the growing axon tips of primary cultured cortical neurons expressing mCherry-α-tubulin at days in vitro (DIV) 3 (Fig. 3*E*). Following KD of Gcap14, the mobile microtubule fraction was reduced by approximately 30% without changing the half-maximum recovery time (Fig. 3 *F*–*I*). These results indicate that functional loss of Gcap14 reduces the dynamic nature of the microtubule without affecting the basic elongation speed.

We further characterized the Gcap14 as a +TIP. We compared the localization and dynamics of Gcap14 relative to EB3, a representative +TIP. In Neuro2a cells and primary cultured cortical neurons, the Gcap14 and EB3 signals were coenriched at the microtubule tips (*SI Appendix*, Fig. S5 *E*–*H*). Moreover, the anterograde movement of Gcap14 and EB3 was evident, and their dynamic movements were largely overlapping in the live cell imaging at the unipolarized microtubule-rich axon terminal (Fig. 3*J*). We also attempted to track the Gcap14 signal in the growth cone during neurite outgrowth. Gcap14 signals were enriched when the microtubules were being elongated and decreased in the shortening and pausing periods (*SI Appendix*, Fig. S5*I*). These results support that Gcap14 dynamically tracks the microtubule plus end displaying a property of the +TIP.

### Gcap14 Recruits Ndel1 to Microtubule Plus Ends.

**Fig. 4.**
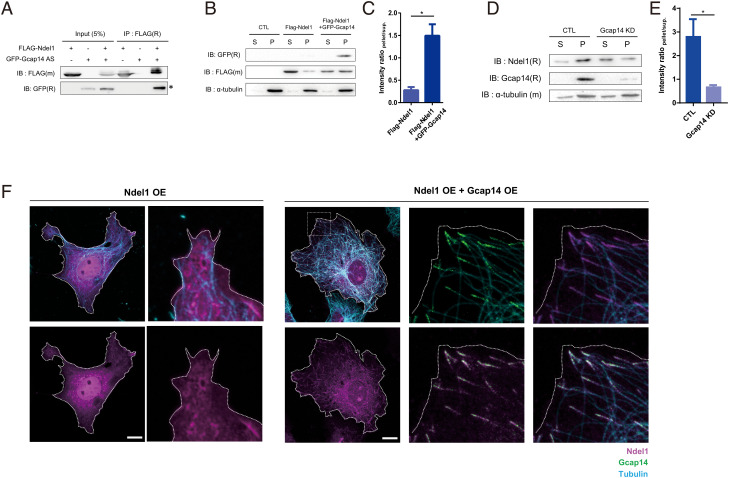
Gcap14 physically interacts with Ndel1 and recruits Ndel1 to the microtubule plus end. (*A*) Coimmunoprecipitation of GFP–Gcap14 with Flag–Ndel1. The lysates were applied to anti-Flag immunoprecipitation and analyzed by immunoblotting. IP; immunoprecipitation. (*B* and *C*) Microtubule cosedimentation assay for Gcap14 and Ndel1 in HEK293 cells. Polymerized tubulin in the pellet fraction (P) and nonpolymerized tubulin in the supernatant (S) were subjected to western blotting (*B*). Quantification of the relative protein level of Ndel1 upon Gcap14 coexpression in the pellet fraction (*C*) (Student’s *t* test, *P = 0.0337). (*D*) Microtubule cosedimentation assay for Ndel1 under Gcap14 KD. (*E*) Quantification of the relative protein level of Ndel1 upon Gcap14 KD in the pellet fraction (*D*) (*F*) Representative images of COS7 cells for observing localization change of Ndel1 upon Gcap14 expression. (The scale bars represent 20 μm.) Data information: Error bars are reported as ± SEM. *P < 0.05, **P < 0.01, ***P < 0.001; n.s., not significant by Student’s *t* test.

In narrowing down the predicted interaction partners of Gcap14 for further study, we utilized the reported functions in the literature, gene ontology criteria (36), and expression profile in the developing brain (Allen Brain Atlas, THE HUMAN PROTEIN ATLAS). First, we focused on the candidates with functions including neurodevelopment, neuronal migration, cytoskeleton, and microtubule (*SI Appendix*, Fig. S6*A*). Among the six candidates from this analysis, DYNLL1/2, which is a component of the dynein complex, and Ndel1 showed positive physical interaction in co-IP experiments (*SI Appendix*, Fig. S6 *C*–*G*). Moreover, Gcap14 and Ndel1 showed very similar expression profile over the neurodevelopment with significant upregulation of expression in the late embryonic stage (*SI Appendix*, Fig. S6*B*). The dynein complex is a microtubule minus-end-directed motor protein that does not correspond to our Gcap14 data. In the subsequent interactome analysis, we identified Ndel1 as a potential interaction partner that is likely to be functionally linked to Gcap14 in the regulation of microtubule dynamics (20, 28, 29). To determine whether Ndel1 physically interacts with Gcap14 in mammalian systems, we performed a coimmunoprecipitation assay using HEK293 cells expressing Flag–Ndel1 or GFP–Gcap14 and confirmed their complex formation (Fig. 4*A*).

To examine the potential role of Gcap14 and Ndel1 in microtubule dynamics, we performed a microtubule cosedimentation assay. Remarkably, the expression of Gcap14 led to an increase in the cosedimentation of Ndel1 with the microtubule (Fig. 4 *B* and *C*). Additionally, we performed a microtubule cosedimentation assay on endogenous Ndel1 fraction under the Gcap14 KD condition. The KD of Gcap14 led to a decrease in the cosedimentation of Ndel1 with the microtubule (Fig. 4 *D* and *E*). In the subsequent immunocytochemical analyses of COS7 cells, Ndel1 was dispersed in the cytoplasm. However, when coexpressed with Gcap14 (Fig. 4*F*), the primary localization of Ndel1 switched to microtubules. Therefore, Gcap14 physically interacts with Ndel1, recruiting Ndel1 to the microtubule plus ends.

**Fig. 5.**
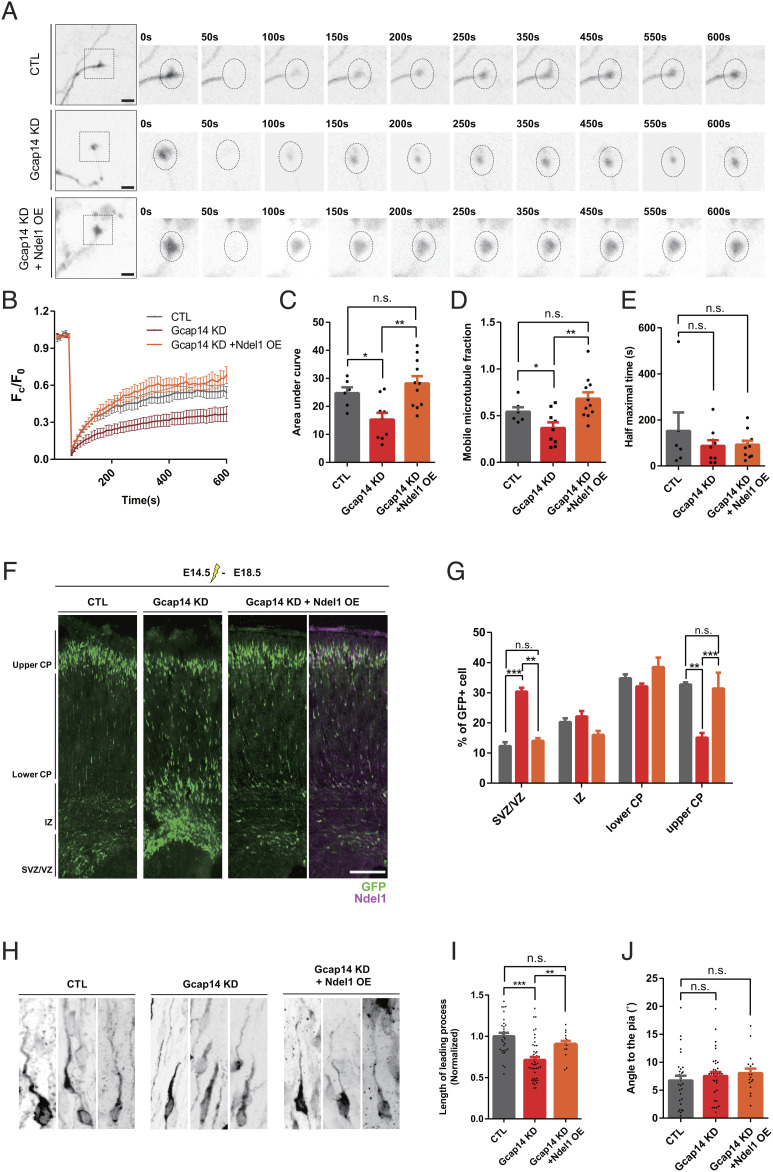
Ndel1 recovers defects of microtubule dynamics and neuronal migration caused by Gcap14 deficiency. (*A*) Representative time-lapse images of the FRAP analysis at the axon tips of primary cultured cortical neurons expressing mCherry-α-tubulin. The dashed circle indicates the ROI used for bleaching. Bleaching was achieved through stimulation with a 10% 568 nm laser for 10 s. (The scale bars represent 10 μm (n > 6). (*B*–*E*) Quantification of time-dependent fluorescence recovery (*B*). Comparisons of the area under FRAP curves (*C*) (one-way ANOVA, *P = 0.0339 CTL versus Gcap14 KD, ***P = 0.0009 Gcap14 KD versus Gcap14 KD+Ndel1 OE). Comparisons of the fraction of mobile microtubule based on the level of eventual fluorescence recovery (*D*) (one-way ANOVA, *P = 0.0282 CTL versus Gcap14 KD, ***P = 0.0001 Gcap14 KD versus Gcap14 KD+Ndel1 OE), and the average half-maximal time of fluorescence recovery (*E*) (no significant changes were observed, *P* > 0.05). (*F*) Representative image of the brain of mouse embryos that were electroporated in utero with shRNA plasmids at E14.5 and analyzed at E18.5 (control, n = 3 brains; Gcap14 shRNA, n = 3 brains; Gcap14 shRNA+Ndel1 OE, n = 3 brains). Coronal sections containing transfected GFP-positive neurons of the cortex (green). (The scale bar represents 100 μm). (*G*) Quantitative analyses of the distribution of transfected cells (one-way ANOVA, SVZ/VZ, ****P < 0.0001 CTL versus Gcap14 KD, ***P = 0.0002 Gcap14 KD versus Gcap14 KD+Ndel1 OE; upper CP, *P = 0.0186 CTL versus Gcap14 KD, *P = 0.0258 Gcap14 KD versus Gcap14 KD+Ndel1 OE). (*H*) Morphological analyses of migrating neurons (control, n = 30 cells; Gcap14 shRNA, n = 45 cells; Gcap14 shRNA+Ndel1 OE, n = 14 cells). (*I* and *J*) Quantification of the length of leading processes (*I*) (one-way ANOVA, ****P < 0.0001 CTL versus Gcap14 KD, *P = 0.0199 Gcap14 KD versus Gcap14 KD+Ndel1 OE). Quantification of the angle of the leading process deviated from the closest connecting line from the cell soma to the pia (*J*) (one-way ANOVA, no significant changes were observed, *P* > 0.05). Data information: Error bars are reported as ± SEM. *P < 0.05, **P < 0.01, ***P < 0.001; n.s., not significant by one-way ANOVA.

To understand the significance of this interaction, we ectopically express Ndel1 in neurons depleted of Gcap14 in vitro and in vivo. Using FRAP to examine microtubule dynamics in the axon tip of primary cultured cortical neurons, where microtubules play critical roles in growing neuronal processes, we found that the expression of Ndel1 effectively rescued the fluorescence recovery suppressed by Gcap14 KD (Fig. 5 *A*–*E*). Moreover, the delay in neuronal migration of Gcap14 KD neurons was also effectively rescued by Ndel1 coexpression in utero (Fig. 5 *F* and *G*). Whereas Gcap14 KD neurons remained in the SVZ/VZ with fewer neurons in the upper CP, Ndel1-coexpressing KD cells mostly migrated into the upper CP. The length of the leading processes was also recovered in these neurons (Fig. 5 *H*–*J*). These results indicate that Gcap14 and Ndel1 cooperate to regulate axonal microtubule dynamics and neuronal migration.

### The Gcap14–Ndel1 Complex Participates in Functional Links between the Microtubule and Actin Filaments.

**Fig. 6.**
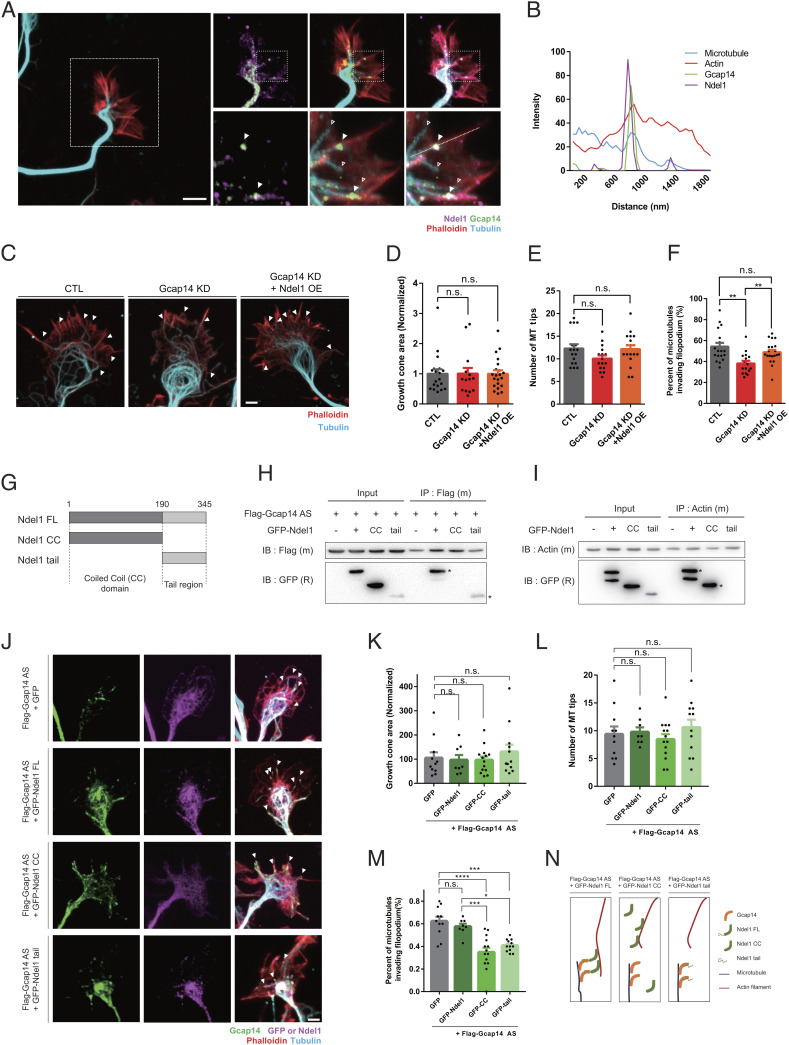
The Gcap14–Ndel1 complex localizes at the microtubule–actin contact sites and bridges microtubules to actin filaments. (*A*) Representative image for localization of Gcap14 and Ndel1 at the interface between actin filaments and microtubules in growth cones. Primary cortical neuron stained for Gcap14 (green), Ndel1 (magenta), F-actin (red), and microtubule (cyan) showing colocalization of Gcap14 and Ndel1 between a single microtubule and actin filament bundle. Filled arrowheads indicate a single microtubule-interfacing actin filament bundle. Open arrowheads indicate the microtubule tip without interaction with the actin filament bundle. (The scale bar represents 20 μm.) (*B*) Quantification of the intensity of the fluorescence along the filopodium along the dotted line in (*A*). (*C*) Representative image for protrusion of the microtubule into the filopodium of the growth cone. The arrowheads indicate the alignment sites between a single microtubule and actin filament bundle. (The scale bars represent 20 μm.) (*D*–*F*) Quantification of the area of the growth cone (*D*), number of microtubule tips (*E*), and the fraction of microtubule of invading filopodium (*F*) (one-way ANOVA, ***P = 0.0009 CTL versus Gcap14 KD, *P = 0.0261 Gcap14 KD versus Gcap14 KD+Ndel1 OE). (*G*) Schematic diagram of the constructs used to map the interaction between Ndel1 and Gcap14 or actin. (*H* and *I*) Coimmunoprecipitation of GFP–Ndel1 fragments with Flag–Gcap14 or actin. The lysates were applied to anti-Flag or anti-actin immunoprecipitation and analyzed by immunoblotting. (*J*) Representative image for protrusion of the microtubule into the filopodium at the growth cone. The arrowheads indicate the alignment sites between a single microtubule and actin filament bundle. (The scale bars represent 20 μm.) (*K*–*M*) Quantification of the growth cone area (*D*), number of microtubule tips (*E*), and the fraction of microtubule of invading filopodium (*F*) (one-way ANOVA, ****P < 0.0001 CTL versus GFP–CC, ***P = 0.0003 CTL versus GFP-tail, ***P = 0.0003 GFP–Ndel1 versus GFP–CC, *P = 0.0126 GFP–Ndel1 versus GFP-tail). (*N*) Schematic diagrams of the roles of the Gcap14–Ndel1 complex in microtubule–actin crosstalk. Data information: Error bars are reported as ±SEM. *P < 0.05, **P < 0.01, ***P < 0.001; n.s., not significant by Student’s *t* test and one-way ANOVA.

At the leading edge of migrating cells, microtubules regulate the protrusive activity of the actin network by modulating actin nucleation and polymerization (37). Conversely, actin guides microtubule growth toward the leading edge and regulates microtubule stability (17, 38, 39). It has been shown that Ndel1 not only regulates microtubule dynamics but also increases the filamentous actin pool, hinting at potential regulatory roles at the interface between two major cytoskeletal networks (27, 28, 31). Therefore, we examined the potential involvement of the Gcap14–Ndel1 complex in microtubule–actin crosstalk. We focused on the growth cone of cortical neurons, where the actin–microtubule crosstalk takes place, and microtubule bundles are aligned with actin filament bundles, forming an essential structural basis for axon specification and filopodia formation (18, 19, 40). The Gcap14–Ndel1 complex was located at the microtubule plus end that aligned with the actin filament bundle invading the filopodia (Fig. 6 *A* and *B*). For quantitative analysis, we categorized the modes of microtubule–actin associations into three groups: “no contact,” “intersection,” and “alignment” (*SI Appendix*, Fig. S7 *A* and *B*). The Gcap14–Ndel1 complex was predominantly associated with the alignment group, showing little “no contact” and intersection patterns (*SI Appendix*, Fig. S7*C*).

To assess the functions of Gcap14 and Ndel1 in the microtubule–actin association, we mediated the expression of Gcap14 or Ndel1 in primary cultured cortical neurons and examined the axon growth cone at DIV3 (Fig. 6*C*). Individual microtubules in control cells protrude into the actin-rich transition region and grow along with actin filament bundles. However, in the Gcap14-deficient growth cones, the fraction of microtubule invading filopodium decreased without detectable changes in the area of the growth cone and the number of microtubule tips (Fig. 6 *D*–*F*). We consistently found that Ndel1 recovered the protrusion of microtubules into the filopodium. These results indicate that Gcap14 and Ndel1 cooperate to regulate microtubule–actin crosstalk in the growth cone.

To further characterize the function of the Gcap14–Ndel1 complex as a cross-linker of microtubule and actin filament, we attempted to narrow down the region of Ndel1 required for bridging microtubule and actin filament. We utilized the CC and tail fragments of Ndel1 in a series of coimmunoprecipitation experiments (Fig. 6*G*). We found that the Gcap14 physically interacted with the tail region of Ndel1, whereas the actin interacted with the CC fragment of Ndel1 (Fig. 6 *H* and *I*). We observed that the full-length and tail fragment signals were well overlapped with Gcap14 at the microtubule tip region, and the Ndel1 CC fragment signals were overlapped with actin signal without microtubule plus end localization (*SI Appendix*, Fig. S7 *D*–*H*). Interestingly, Ndel1 CC and tail region reduced the microtubule fraction invading filopodium without changes in the growth cone area and the number of microtubule tips (Fig. 6 *J*–*M*). These Ndel1 mutants disrupted the microtubule–actin crosstalk working as dominant-negative forms (Fig. 6*N*). Taken together, these data demonstrate that the Gcap14–Ndel1 complex functions as a bridge between microtubule and actin filaments at growth cones.

The defects were extended to the formation of filopodia and neurite outgrowth. Gcap14 KD in DIV3 cortical neurons reduced filopodia number (*SI Appendix*, Fig. S8 *A*–*C*) and neurite length (*SI Appendix*, Fig. S8 *D* and *E*). Again, coexpression of Ndel1 restored the filopodia number but not the length of the longest neurites. Moreover, when the projection and ipsilateral branching of the primary cortical neurons were analyzed (IUE at E14.5 and analysis at P7), although Gcap14 KD neurons eventually migrate into the upper CL at P7, the GFP-positive Gcap14-deficient axon fibers seldom passed the corpus callosum (*SI Appendix*, Fig. S9*A*) and formed a relatively sparse network of ipsilateral axon branches in layer V (*SI Appendix*, Fig. S9 *B*–*D*). Therefore, the migration delay accompanies by defective ipsilateral branching and contralateral cortical projection, implying critical roles of Gcap14 in neuronal migration and associated neurodevelopmental processes. In addition, Ndel1 coexpression reversed the ipsilateral branching defect in Gcap14 KD neurons without affecting the axon projection defects, suggesting that Ndel1 is functionally associated with the roles of Gcap14 in neuronal maturation, including filopodia formation and axon branching.

## Discussion

**Fig. 7.**
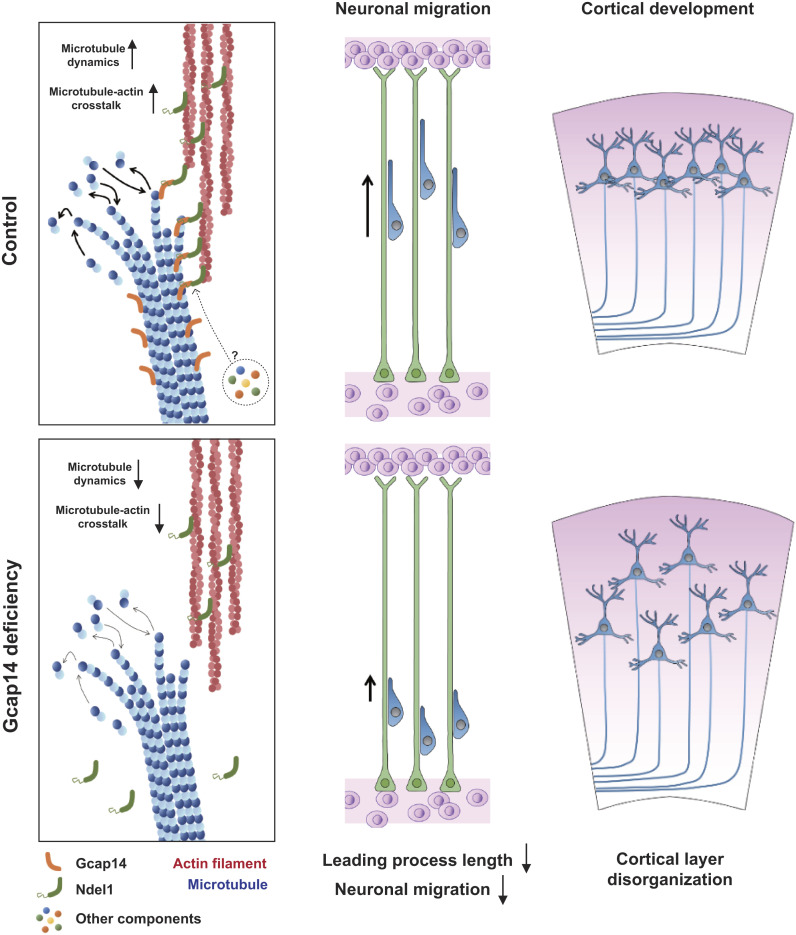
The Gcap14 localizes at microtubule plus end, recruiting Ndel1 to the tip of the microtubule. Through the interaction of the Gcap14–Ndel1 tail region and actin–Ndel1 CC domain, Gcap14 and Ndel1 participate in microtubule–actin crosstalk. The Gcap14–Ndel1 complex regulates the microtubule dynamics and cross-links between the microtubule and actin. The microtubule–actin crosstalk engages in the processes of neuronal migration including the formation of leading processes and the speed of the migration. In Gcap14 deficiency, Ndel1 is restricted to localize at the microtubule plus end. The neuronal migration is impaired with the shortened leading process, thereby causing disorganization of the CL. Consequently, Gcap14 acts as a microtubule plus-end-tracking protein for regulating cortical development.

In this study, we propose a molecular process underlying neuronal migration and maturation mediated by the Gcap14–Ndel1 complex. The core of the process engages in cytoskeletal crosstalk at the tip of microtubules in the growth cone area, revealing a role for Gcap14 as a +TIP (Fig. 7).

It is known that +TIPs play critical roles in several regulatory mechanisms of microtubule dynamics. The XMAP215 family has polymerase activity that promotes faster microtubule elongation with enhanced stability (41, 42). CLASP family members, such as Stu1 and Cls1p, may reduce catastrophe and increase rescue by tethering unpolymerized tubulin to the microtubule (43–45). In particular, an isolated Stu1- Tumor Overexpressed Gene (TOG) domain modulates only the frequency of catastrophe and rescue without changing the microtubule elongation rate (45). Given that the deficiency of Gcap14 reduced the mobile microtubule fraction without changing the half-maximal time, as shown in our FRAP analysis (Fig. 3 *E*–*I*), and that Gcap14 also directly binds to unpolymerized tubulin and microtubule (34), it is likely that Gcap14 takes part in tethering tubulins to microtubules to enhance the pool of dynamic microtubules in the multiple steps of neurodevelopment.

In our study, the Gcap14 recruits Ndel1 at the plus end to bridge the microtubule to the actin cytoskeleton. Ndel1 targets dynein to the plus end of the microtubule, which in turn recruits LIS1 to dynein (29). The Ndel1–dynein–LIS1 complex regulates microtubule self-organization, microtubule dynamics, and dynein-mediated cargo transport (28, 29). An atypical protein kinase C–Aurora-A–NDEL1 pathway regulates microtubule organization during neurite extension (46). On the contrary, Ndel1 is recruited by TARA toward filamentous actin in the cell periphery and enhances the filamentous actin dynamics regulated by the TARA–DYRK2–GSK3β complex (27, 31). The physical bridge in the microtubule–actin filament has a crucial meaning in cytoskeletal remodeling. Microtubule–actin crosstalk in a cell is vital for guiding microtubule growth by bundles of the actin filament and actin filament organization. Especially, as shown in the study by Lopez et al. (47), a simple artificial linkage between actin and microtubule is sufficient to enhance the efficiency of microtubule organization. Therefore, although the mode of crosstalk between microtubule and actin filament achieved by the Gcap14–Ndel1 complex still demands further clarification, it is relatively evident that Gcap14 not only regulates microtubule dynamics at the end of the microtubule as a +TIP but also mediates microtubule–actin crosstalk in complex with actin-associated Ndel1 in the growth cone regions. Gcap14 may play a role in the stability of the cytoskeleton regulatory complex composed of Ndel1 and the functional interacting proteins.

We also observed that enhanced Ndel1 coexpression reverses defects in microtubule dynamics and neuronal migration caused by Gcap14 deficiency. Although these observations strengthen the notion that Gcap14 and Ndel1 function in an overlapping pathway, the mechanistic explanation is complex. The +TIPs cooperatively bind to the microtubule plus end, forming a +TIPs network (48). For example, CLIP-170, APC, and Tip2p accumulate at the microtubule plus end by binding to EB1 (49, 50). The multipartite interactions of +TIPs are likely to contribute to their synergistic recruitment to the microtubule plus end. Likewise, under a Gcap14-deficient condition, we supposed that an excessive level of Ndel1 might increase the probability of interacting with other +TIPs, such as the dynein–LIS1 complex or TACC3 (28, 29, 51). These interactions may help Ndel1 bypass the functional deficiency of Gcap14. Alternatively, a small amount of residual Gcap14 in KD cells is sufficient to recover defects with ectopically overexpressed Ndel1. The protein levels of Gcap14 provide a measure for the average KD (*SI Appendix*, Fig. S2), but excessive Ndel1 increases the chances of interaction with residual Gcap14. This Gcap14–Ndel1 complex may address defects caused by Gcap14 deficiency. Understanding how the expression of Ndel1 contributes to the recovery of defects by Gcap14 deficiency is a significant question for future works.

In our initial histological analysis of *Gcap14* KO mice, we discovered that Gcap14 deficiency causes slight but significant thickening of the neocortex. This phenomenon appears unusual in that defects in neuronal migration often cause a reduction in cortical thickness (52, 53). However, several studies have also reported an association between abnormal neuronal migration and increased cortical thickness. Multiple cases of enlarged cortices have been reported. For example, mice expressing a constitutively active mutant *Fgfr3* allele demonstrated increased cortical thickness due to the increased proliferation of progenitor cells. The fetal brain of LPS-treated mouse embryos also showed migration failure and an increase in fetal cortical thickness due to microglial activation. Alternatively, enlarged neuronal soma size is suggested to be a factor in cortical thickening. Tuberous sclerosis complex 2 KO mice showed cortical thickening with enlarged soma without changes in the number of neurons (54). Along the line, astroglial phosphatase and tensin homolog (PTEN) loss also causes macrocephaly and cellular disorganization in association with increased neuronal soma size (55, 56). Intriguingly, Gcap14-deficient neurons also exhibited a significant increase in soma size (*SI Appendix*, Fig. S3), hinting at a potential explanation of the thickened cortex of *Gcap14* KO mice.

The interstitial deletion of chromosome 10q22-23, in which the *Gcap14* gene localizes (10q23.1), is a well-documented chromosomal abnormality associated with 10qdel syndromes, such as Cowden syndrome, Bannayan–Riley–Ruvalcaba syndrome, and juvenile polyposis syndrome (57). Patients with 10qdel syndrome suffer from mild developmental delay, mild dysmorphism, and cognitive retardation. Moreover, 10qdel syndrome is accompanied by macrocephaly with symptoms of autism and epilepsy (58–60). The genes encoding PTEN and bone morphogenetic protein receptor type 1A in this genomic region have been suggested as the culprits, but the molecular basis of 10qdel syndrome has not been clearly elucidated (61, 62). Given that Gcap14 deficiency causes neurodevelopmental defects with thickening of the cortex reminiscent of macrocephaly seen in 10qdel syndromes, it is intriguing to speculate the contribution of Gcap14 deficiency to the 10qdel syndromes. Therefore, investigation of the neurodevelopmental function of Gcap14 as a microtubule +TIP might elucidate the pathobiology of human 10qdel syndrome.

## Materials and Methods

### Plasmids and Antibodies.

For the mouse Gcap14 construct, isoforms of the mGcap14 coding sequence (NM_001347502.1, NM_028407.4) were amplified from the complementary DNA (cDNA) library obtained from the mouse brain and inserted into pEGFP-C3. For the Ndel1 construct, a mouse Ndel1 coding sequence from the cDNA library was amplified and inserted into pFLAG-CMV2. The mCherry-α-tubulin construct was purchased from Addgene (Watertown, MA, USA). The oligonucleotide sequence of the Gcap14 shRNA was 5′-GCATGACGGAAGTGGATCAT-3′. These oligonucleotides were annealed and ligated into the pLentiLox3.7 vector using the HpaI and XhoI sites.

Anti-GFP rabbit polyclonal (A11122) antibody was purchased from Molecular Probes (Eugene, OR, USA). Anti-GFP mouse monoclonal (sc-9996) was purchased from Santa Cruz Biotechnology (Dallas, TX, USA). Anti-FLAG M2 (F1804) antibody was purchased from Sigma-Aldrich (Saint Louis, MO, USA). Anti-FLAG rabbit polyclonal antibody (PA1-984B) was purchased from Thermo Fisher Scientific (Waltham, MA, USA). Anti-Gcap14 rabbit polyclonal (ab122393), anti-CTIP2 rat monoclonal (ab18465), anti-TBR1 rabbit polyclonal (ab31940), and anti-SATB2 mouse monoclonal (ab51502) were purchased from Abcam (Cambridge, UK). Anti-Ndel1 rabbit polyclonal (AB-2235821) and anti-α-tubulin mouse monoclonal antibodies (sc-32293) were purchased from Proteintech (Rosemont, IL, USA).

### Generation of *Gcap14* KO Mice.

*Gcap14* KO mice were generated by Macrogen (Seoul, South Korea). To generate male *Gcap14* mutants, a female pup for a 14333bp deletion, which generated a premature stop codon in *Gcap14* exon 4, was cross-bred with a C57BL/6N wild-type male mouse. The mice were interbred and maintained under Specific-pathogen-free conditions. All animal procedures were approved by the Pohang University of Science and Technology’s Institutional Animal Care and Use Committee. Briefly, C57BL/6N female mice were treated with pregnant mare serum gonadotropin and human chorionic gonadotropin. After 48 h, the female mice were mated with C57BL/6N male mice. The next day, female mice containing vaginal plugs were killed, and the fertilized embryos were harvested. A mixture of SpCas9 protein tagged with a nuclear localization signal and guide RNAs (gRNAs) targeting *Gcap14* exons 1 and 3 was microinjected into one-cell embryos. The microinjected embryos were incubated at 37 °C for 1 or 2 h, after which they were transplanted into the oviducts of pseudopregnant recipient mice.

### Cell Culture and Transfection.

HEK293 and COS7 cells were cultured in Dulbecco's Modified Eagle Medium (DMEM) (HyClone, South Logan, UT, USA) supplemented with 10% (v/v) fetal bovine serum (Gibco, Gaithersburg, MD, USA) and 1% penicillin/streptomycin (Gibco). All cell lines were authenticated using the Short tandem repeat profiling method and tested negative for mycoplasma contamination. All cells were transfected using a transfection reagent, either polyethylenimine (23966-2, Polysciences, Inc., Warrington, PA, USA) (63) or Lipofectamine 2000 (Thermo Fisher Scientific).

Primary cultures of cortical neurons were established by isolating E15.5 C57BL/6 mouse embryo cortical tissues in Hanks' Balanced Salt Solution (HBSS) (Gibco) and dissociating tissues in 0.25% trypsin (Sigma-Aldrich) and 0.1% DNase I (Sigma-Aldrich) for 15 min at 37 °C. The cells were resuspended in a neurobasal medium (Gibco) supplemented with 10 mM HEPES pH 7.4% and 10% (v/v) horse serum for a final cell concentration of 4.0 × 10^5^ cells/mL and plated on glass coverslips precoated with poly-D-lysine and laminin. After 2 h of plating, the cell medium was replaced with a neurobasal medium containing 2 mM glutamine, 2% (v/v) B27 supplement (Gibco), and 1% (v/v) penicillin/streptomycin. The neurons were transfected with Lipofectamine 2000, and the medium was replaced with the culture medium 2 to 4 h after transfection.

### Coimmunoprecipitation.

The cultured cells were homogenized in ELB lysis buffer (50 mM Tris, pH 8.0, 250 mM NaCl, 0.1% NP-40, 5 mM EDTA, 5 mM glycerol-2-phosphate, 2 mM sodium pyrophosphate, 5 mM NaF, 2 mM Na_3_VO_4_, 1 mM DTT, EDTA-free protease inhibitor cocktail) and precleared by centrifugation for 10 min at 12,000 × g. For immunoprecipitation, the lysates were incubated with antibodies (1 μg) on a rocking platform overnight at 4 °C. Then, 25 μL of 10% protein-A agarose (Roche, Basel, Switzerland) resuspended in the same lysis buffer was added and incubated with gentle shaking for an additional 2 to 3 h at 4 °C. The precipitates were washed three times with lysis buffer and resuspended in SDS sample loading buffer. The precipitates were subjected to anti-Gcap14, anti-Ndel1, anti-GFP, or anti-FLAG immunoblotting.

### Microtubule Cosedimentation Assay.

The microtubule cosedimentation assay was carried out as described previously with modifications (64, 65). Briefly, cultured HEK293 cells were lysed in microtubule-stabilizing buffer (100 mM PIPES [pH 6.8], 0.1% NP40, 5 mM MgCl2, 2 mM EGTA, 100 mM NaF, 2 M glycerol, and protease inhibitor cocktail) and centrifuged at 20,000 × g for 40 min at 4 °C. The supernatant was treated with 30 μM Taxol and incubated for 30 min at 37 °C, followed by centrifugation at 12,000 × g for 40 min at room temperature. The pellets were resuspended in microtubule-stabilizing buffer, and the supernatant was dissolved in the SDS-PAGE sample buffer and subjected to western blot analysis.

### Immunocytochemistry and Immunohistochemistry.

For immunocytochemistry, cells were fixed with 4% paraformaldehyde in phosphate buffered saline (PBS) or 4% paraformaldehyde and 4% sucrose in PBS for 10 min and washed with PBS three times. The cells were permeabilized with 0.1% Triton X-100 in PBS for 5 min and blocked with 3% bovine serum albumin (BSA) in PBS for more than 30 min. For protein staining, the cells were incubated with primary antibodies diluted in 5% BSA in PBS overnight at 4 °C, washed with PBS three times, and treated with secondary antibodies diluted in 5% BSA in PBS for 2.5 h at room temperature.

For immunohistochemistry, mouse brain slices on glass slides were washed with PBS and fixed with 4% paraformaldehyde in PBS for 20 min. After washing three times with PBS, the cells were permeabilized with 0.5% Triton X-100 in PBS for 10 min and then treated with 5% goat serum blocking solution for 1 h. The cells were incubated in primary antibody diluted in the blocking solution overnight at 4 °C. After washing three times with PBS, the cells were incubated in fluorescent-conjugated secondary antibody diluted in blocking solution for 2 h at room temperature. Tissue slides were washed with PBS and mounted using UltraCruz Aqueous Mounting Medium with DAPI (Cat# sc-24941, RRID: AB_10189288, Santa Cruz Biotechnology).

Cell images were acquired using an FV3000 confocal laser scanning microscope (Olympus, Tokyo, Japan) and processed using ImageJ (Fiji) software (RRID: SCR_002285, National Institute of Health, Bethesda, MD, USA) (66).

### Time-Lapse Live Imaging for FRAP Assay.

Cultured primary cortical neurons (DIV3) expressing mCherry-α-tubulin were imaged at 37 °C with 5% CO_2_ gas supply using an FV3000 confocal laser scanning microscope to investigate microtubule dynamics. Using the FV31S-DT software, a 10-mm radius circular region of interest (ROI) around the cellular process tips for microtubule dynamics was determined. The ROI was photobleached by scanning with a 10% power 568 nm laser and 20ms/pixel scan speed for a total of 10 s. For FRAP analysis, five frames were acquired as prebleach images followed by bleaching, and 55 frames were acquired as postbleach images obtained with 10 s intervals. FRAP results were analyzed using the easyFRAP-web application combined with manual analysis (67).

### IUE.

Pregnant C57BL/6 mice at E14.5 were anesthetized with isoflurane (induction, 3.5%; surgery, 2.5%), and the uterine horns were exposed by laparotomy. Coding sequences of target genes in pCIG2 vectors or shRNA sequences in pLL3.7-EGFP vectors were purified using the EndoFree plasmid maxi kit (Qiagen, Germantown, MD, USA). DNA solution (2.0 µg/µL) mixed with 0.1% Fast Green solution was injected into the bilateral ventricles of the embryo through a pulled microcapillary tube. A tweezer-type electrode containing two disc-type electrodes was placed at an appropriate angle, and electric pulses were administered at 35 V, 50 ms, five times, at 950 ms intervals using an electroporator (Harvard Apparatus, Holliston, MA, USA). After electroporation, embryos were placed back into the mother’s abdomen, the incision was sutured, and the mice were returned to their home cage. The mice were killed at E18.5 or P7, and their brains were fixed with 4% paraformaldehyde with 10% sucrose in PBS for 24 h; dehydrated with 10%, 20%, and 30% sucrose in PBS for more than 24 h; and soaked and frozen in Surgipath FSC22 Clear OCT solution (Leica Biosystems, Richmond, IL, USA). Brain tissue was sectioned using cryostats (Leica Biosystems) with 35 µm thickness, and each section was immediately bound to Superfrost Plus microscope slides (Thermo Fisher Scientific). Brain slice images were acquired using 10× and 20× objective lenses of FV3000 confocal laser scanning microscope (Olympus) with Z-stacks of 0.4 µm intervals.

### Statistical Analysis.

Data were obtained from at least three independent experiments. The number of cells studied is indicated in the figure legends. Data were analyzed using GraphPad Prism 7 software and are presented as the mean ± SEM. Statistical significance was determined using Student’s *t* test or one-way ANOVA. *P* < 0.05 was considered statistically significant.

## Supplementary Material

Appendix 01 (PDF)

## Data Availability

All study data are included in the article and/or *SI Appendix*.
