# Supplementary material for: Gcap14 is a microtubule plus-end-tracking protein coordinating microtubule–actin crosstalk during neurodevelopment
Source: Proc Natl Acad Sci U S A. 2023 Feb 16;120(8):e2214507120. doi: 10.1073/pnas.2214507120 (PMC9974511; doi:10.1073/pnas.2214507120)
Supplement: Supplementary file 1 — Appendix 01 (PDF) [file pnas.2214507120.sapp.pdf]

PNAS

Supporting Information for

Gcap14 is a novel microtubule plus-end-tracking protein coordinating microtubule-actin crosstalk during neurodevelopment.

Author List
Dong Jin Mun[1], Bon Seong Goo[1], Bo Kyoung Suh[1], Ji-Ho Hong[1,2], Youngsik Woo[1], Soo Jeong Kim[1], Seunghyun Kim[1], Su Been Lee[1], Yubin Won[1], Jin Yeong Yoo[1], Eunbyul Cho[1], Eun Jin Jang[1], Truong Thi My Nhung[1], Hong Minh Triet[1], Hongyul An[3], Haeryun Lee[1], Minh Dang Nguyen[4], Seung-Yeol Park[1], Seung Tae Baek[1], Sang Ki Park[1]*

*Correspondence

***Email:** skpark@postech.ac.kr

This PDF file includes:

Figures S1 to S9

Fig S1

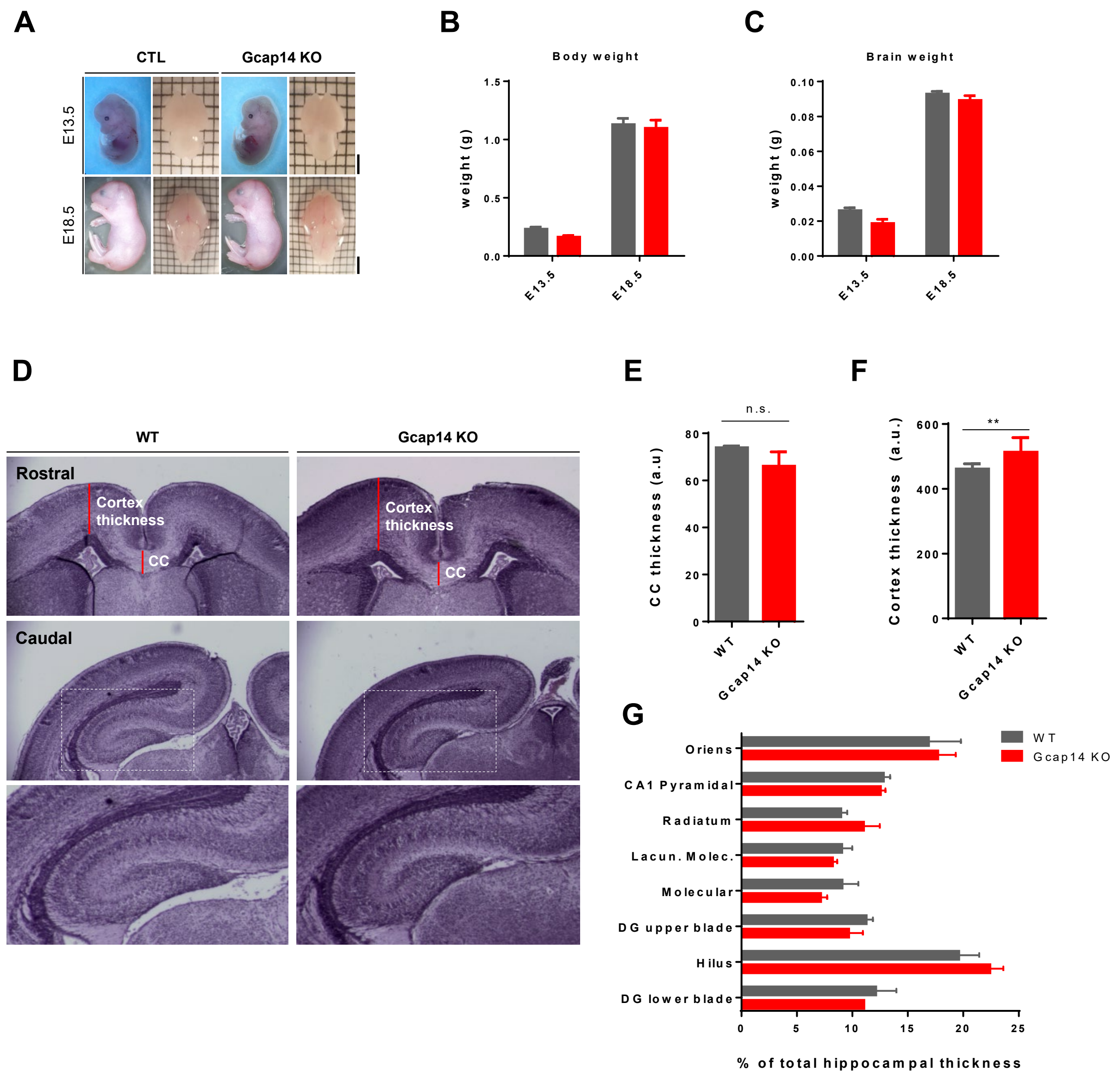

Fig S1.

Morphological changes and Histological analyses of Gcap14 KO embryos. (A) Representative image of the body and brain of WT and Gcap14 KO mice. (B-C) Quantification of body weight and brain weight. The scale bars represent 2mm. (D) Histological analysis of the brain of WT and Gcap14 KO mice at E18.5. (E-G) Quantification of CC thickness, cortex thickness, and percentage of each hippocampal layer thickness. All results are presented as mean ± SEM. (N>3) *p<0.05, **p<0.01, ***p<0.001; n.s., not significant by student's t-test.

Fig S2

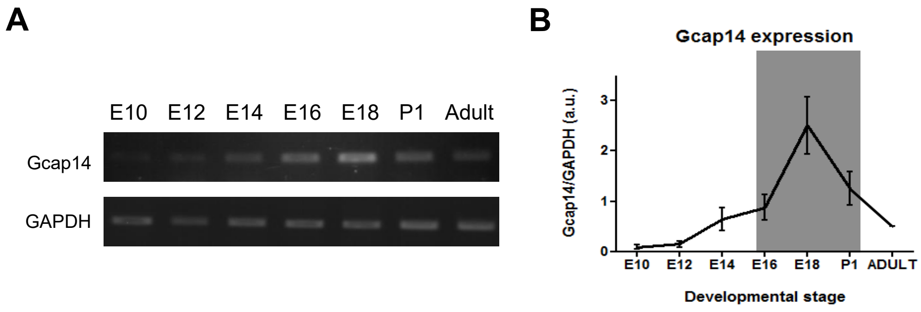

Fig S2.

Gcap14 mRNA profiling. (A) qRT-PCR data for Gcap14 mRNA expression profile had a peak during the perinatal stage. (B) Quantification of Gcap14 mRNA level depending on the developmental period.

Fig S3

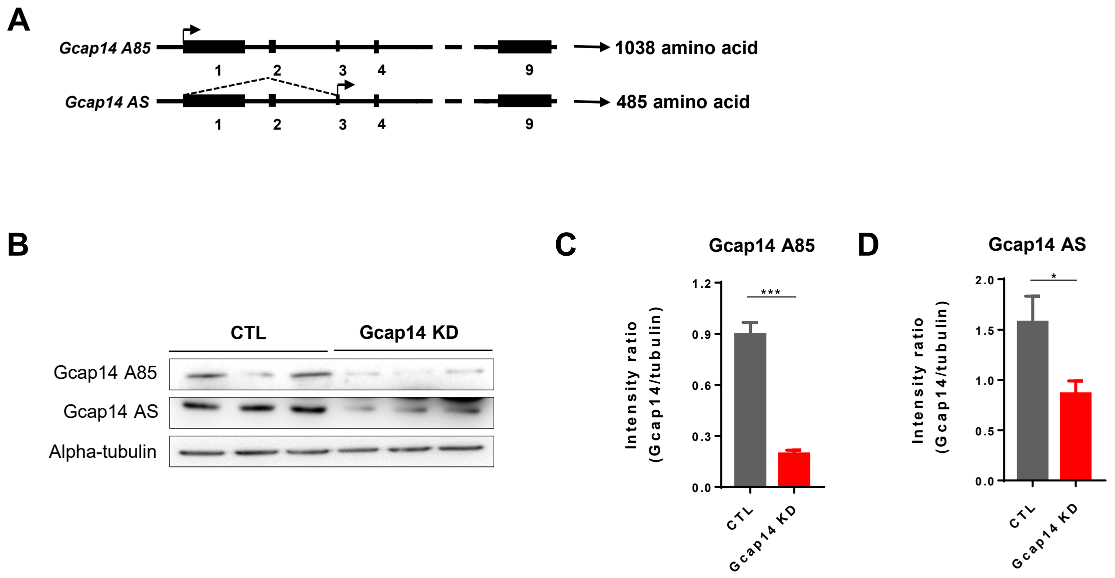

Fig S3.

Gcap14 shRNA reduced Gcap14 protein level. (A) Schematic diagram of the alternative splicing of each Gcap14 isoform. (B) Analysis of Gcap14 shRNA for reducing Gcap14 protein level in N2A cells. (C-D) Quantification of intensity ratio (Gcap14/α-tubulin) are shown. All results are presented as mean ± SEM. *p<0.05, **p<0.01, ***p<0.001; n.s., not significant by student's t-test.

Fig S4

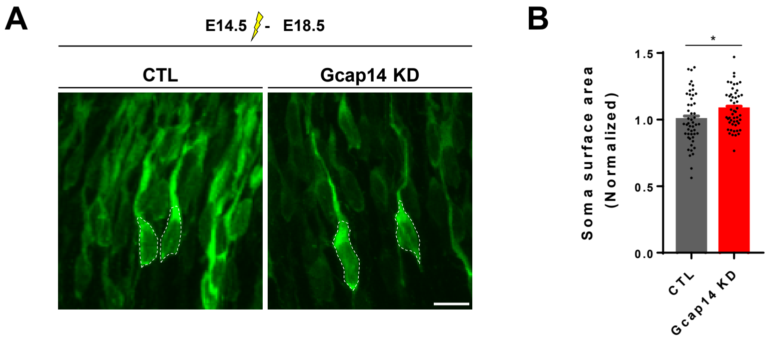

Fig S4.

Soma size in Gcap14-deficient cortical neurons. (A) Representative image of brain of mouse embryos that were electroporated *in utero* with shRNA plasmids at E14.5 and analyzed at E18.5 (control, N=3 brains, n=50 neuron; Gcap14 shRNA, n=4 brains, n=50 neuron). The scale bars represent 10μm.(B) Quantification of soma size are shown. All results are presented as means ± SEM. *p<0.05, **p<0.01, ***p<0.001; n.s., not significant by student's t-test.

Fig S5

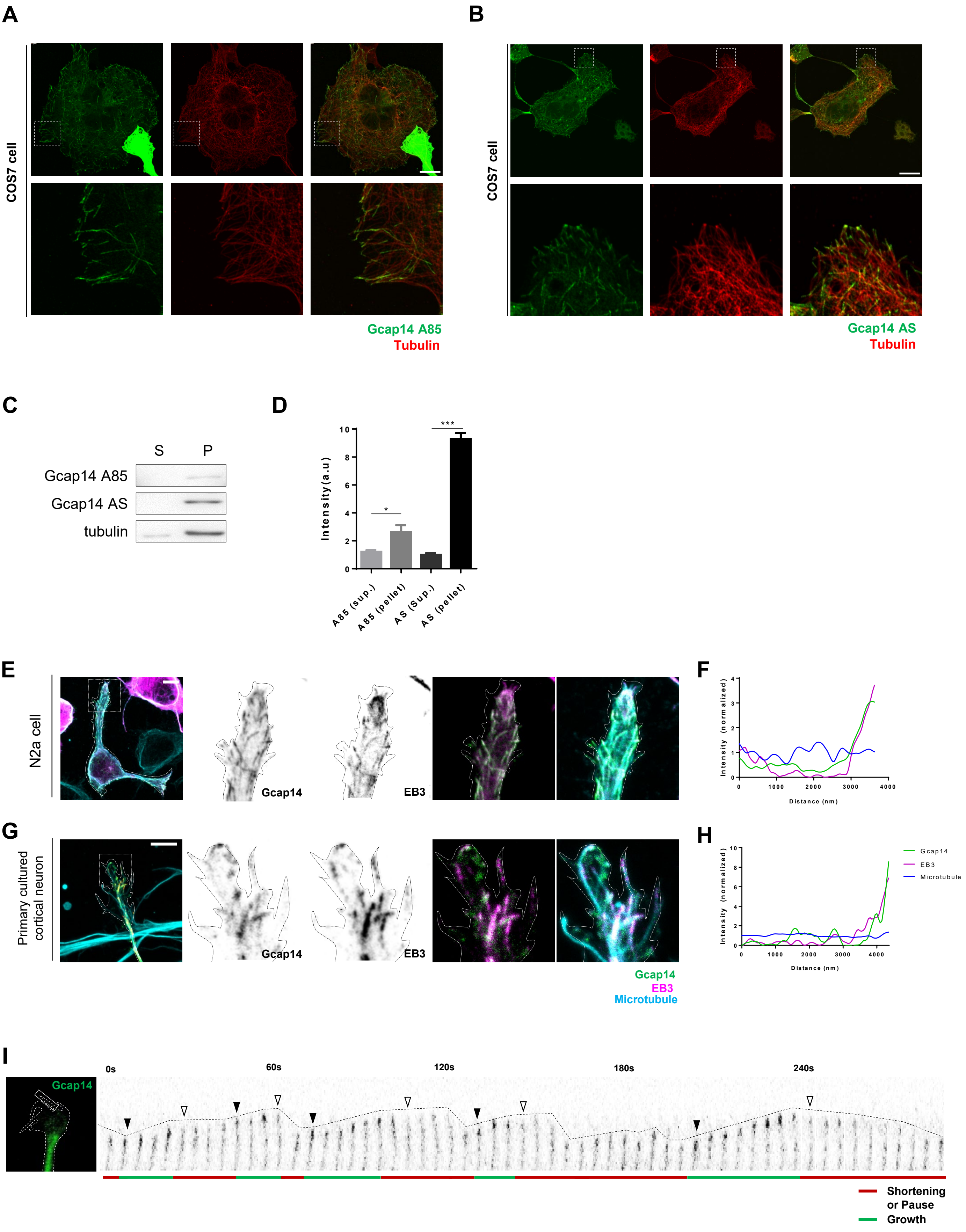
A
COS7 cell
Gcap14 A85
Tubulin
B
COS7 cell
Gcap14 AS
Tubulin
C
S
P
Gcap14 A85
Gcap14 AS
tubulin
D
Intensity(a.u)
*
***
A85 (sup.)
A85 (pellet)
AS (Sup.)
AS (pellet)
E
N2a cell
Gcap14
EB3
F
Intensity (normalized)
Distance (nm)
G
Primary cultured cortical neuron
Gcap14
EB3
Gcap14
EB3
Microtubule
H
Intensity (normalized)
Distance (nm)
Gcap14
EB3
Microtubule
I
Gcap14
0s
60s
120s
180s
240s
Shortening or Pause
Growth

Fig S5

Fig S5.

Gcap14 functions as +TIP and tracks the elongated microtubules. (A-B) Representative image of ectopically expressed Gcap14 A85 and AS in COS7 cells. The scale bars represent 20μm. (C) Microtubule co-sedimentation assay for Gcap14 each isoform in HEK293 cell. Polymerized tubulin in the pellet fraction (P) and non-polymerized tubulin in the supernatant (S) were subjected to western blotting with anti-Gcap14 and anti-α-tubulin antibodies. (D) Quantification of the relative protein level of Gcap14 each isoform in the pellet fraction. (E, G) Immunocytochemical analysis of Flag-Gcap14 and GFP-EB3 localization in the Neuro 2a cell and the primary cultured mouse cortical neuron. Each scale bar represents 10μm (A) and 20 μm (C). (F, H) Quantification of the intensity of the Flag-Gcap14 and GFP-EB3 signals along the dotted line in (E, G). (I) Representative live cell images of the GFP-Gcap14 on the growth cone of cortical neuron. Filled arrowheads indicate a enhanced Gcap14 signal and open arrowheads indicate the weakened Gcap14 signal. All results are presented as mean ± SEM. *p<0.05, **p<0.01, ***p<0.001; NS, not significant by student's t-test.

Fig S6

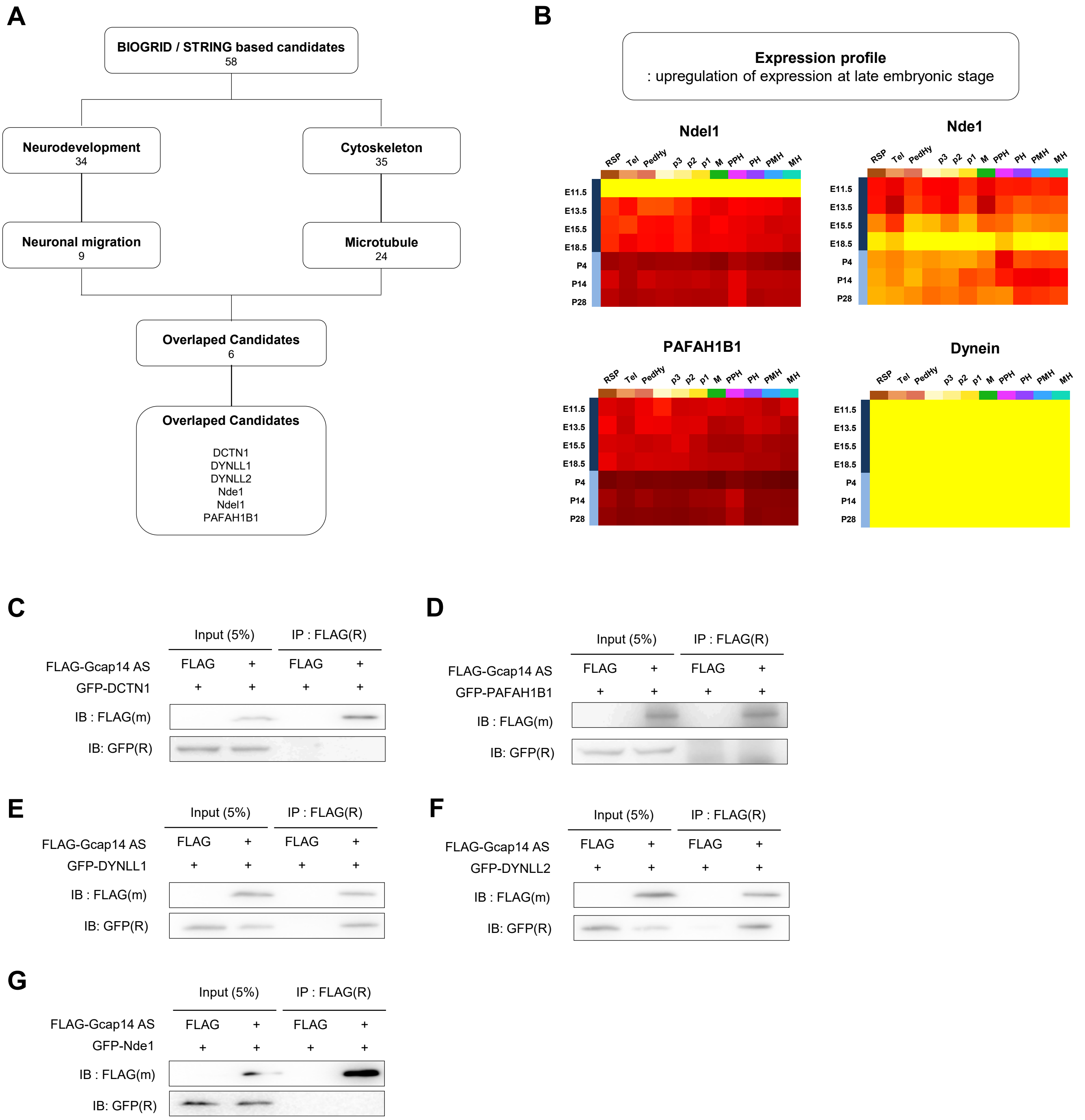

Fig S6.

Ndel1 as a potential interaction partner that is likely to be functionally linked to Gcap14 in the regulation of microtubule dynamics and neuronal migration. (A) The reported function-based algorithm for screening the candidates of functional interaction partner of Gcap14. (B) The *in situ* hybridization data across mouse brain development from Allen Brain Atlas. (C-G) Co-immunoprecipitation of Flag-Gcap14 with GFP-DCTN1, GFP-PAFAH1B1, GFP-DYNLL1, GFP-DYNLL2, GFP-Nde1. The lysates were applied to anti-Flag immunoprecipitation and analyzed by immunoblotting. IP; immunoprecipitation.

Fig S7

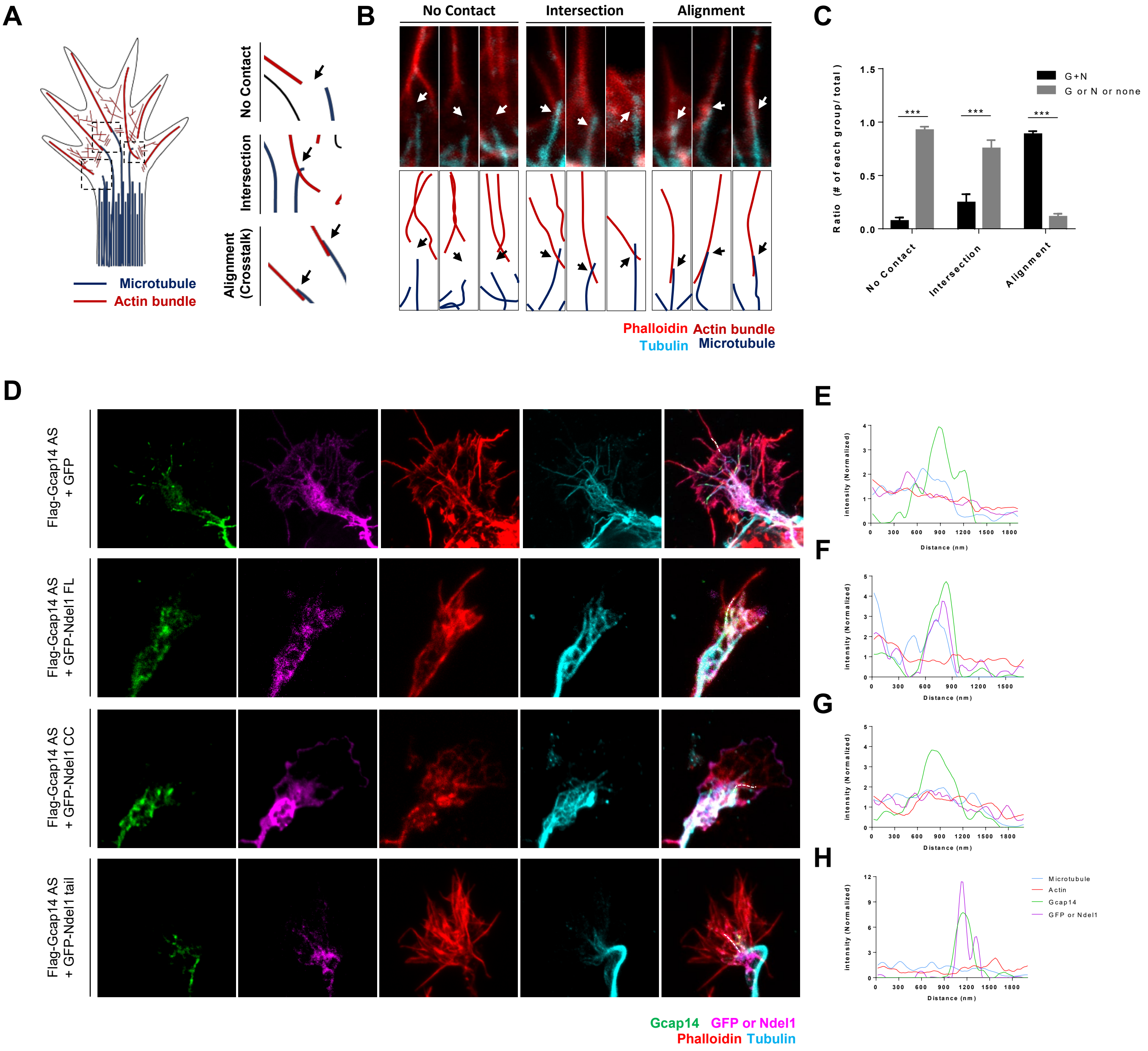

Fig S7.

The function of Gcap14-Ndel1 complex as cross-linker between microtubule and actin filament. (A-B) Types of interaction between a single microtubule and actin filament bundle. Representative images and schematic diagrams of microtubule-actin interactions. (C) Quantitative analysis of Gcap14 and Ndel1 localization relative to types of microtubule and actin filament bundle interaction. (D) Representative image for localization of Gcap14 and Ndel1 mutant at the interface between actin filaments and microtubules in growth cones. (E-H) Quantification of the intensity of the fluorescence along the filopodium along the dotted line in (D). All results are presented as means ± SEM. *p<0.05, **p<0.01, ***p<0.001; NS, not significant by student's t-test.

Fig S8

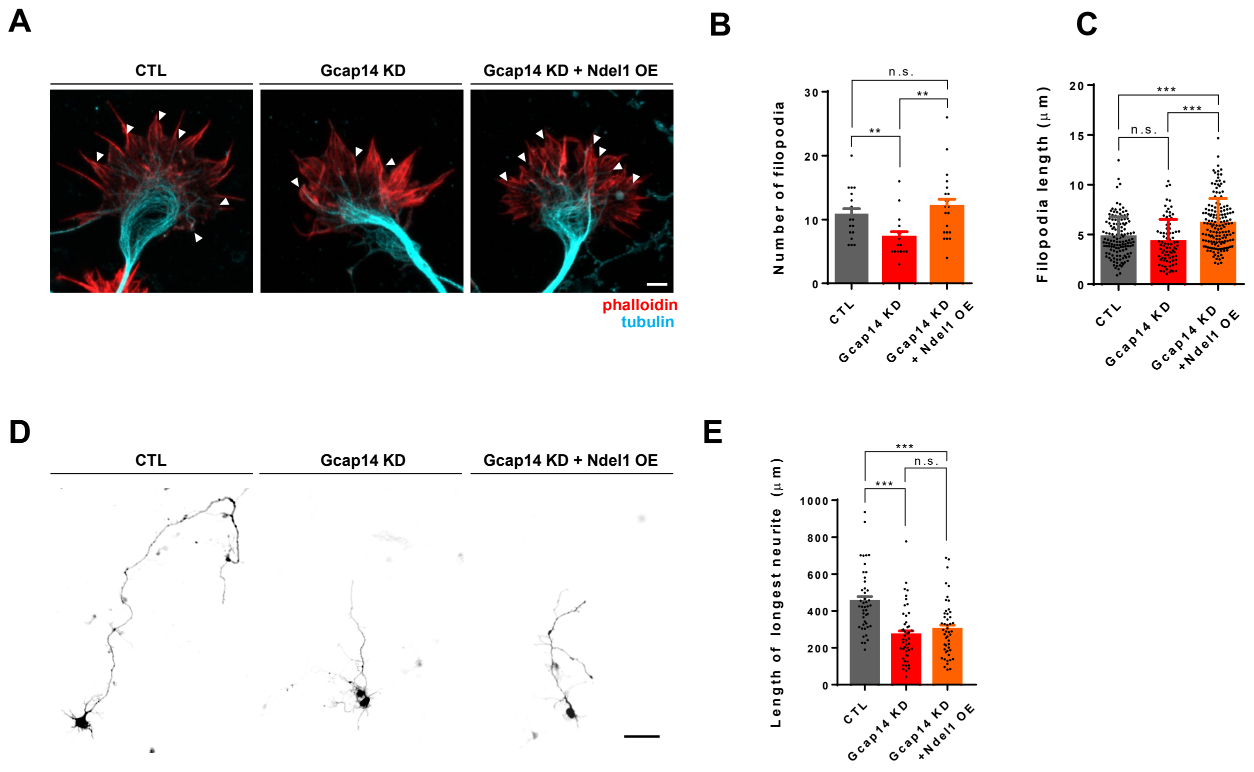

Fig S8.

The effects of Gcap14-Ndel1 complex on filopodia formation and neurite outgrowth. (A) Representative image of filopodia of cultured cortical neuron at DIV3. The scale bars represent 20μm. (B-C) Quantification of filopodia number and length. (D) Representative image of DIV3 primary cultured cortical neurons for measuring the longest neurite length. The scale bars represent 100μm. (E) Quantification of longest neurite length (n>45). All results are presented as mean ± SEM. *p<0.05, **p<0.01, ***p<0.001; NS, not significant by one-way ANOVA

Fig S9

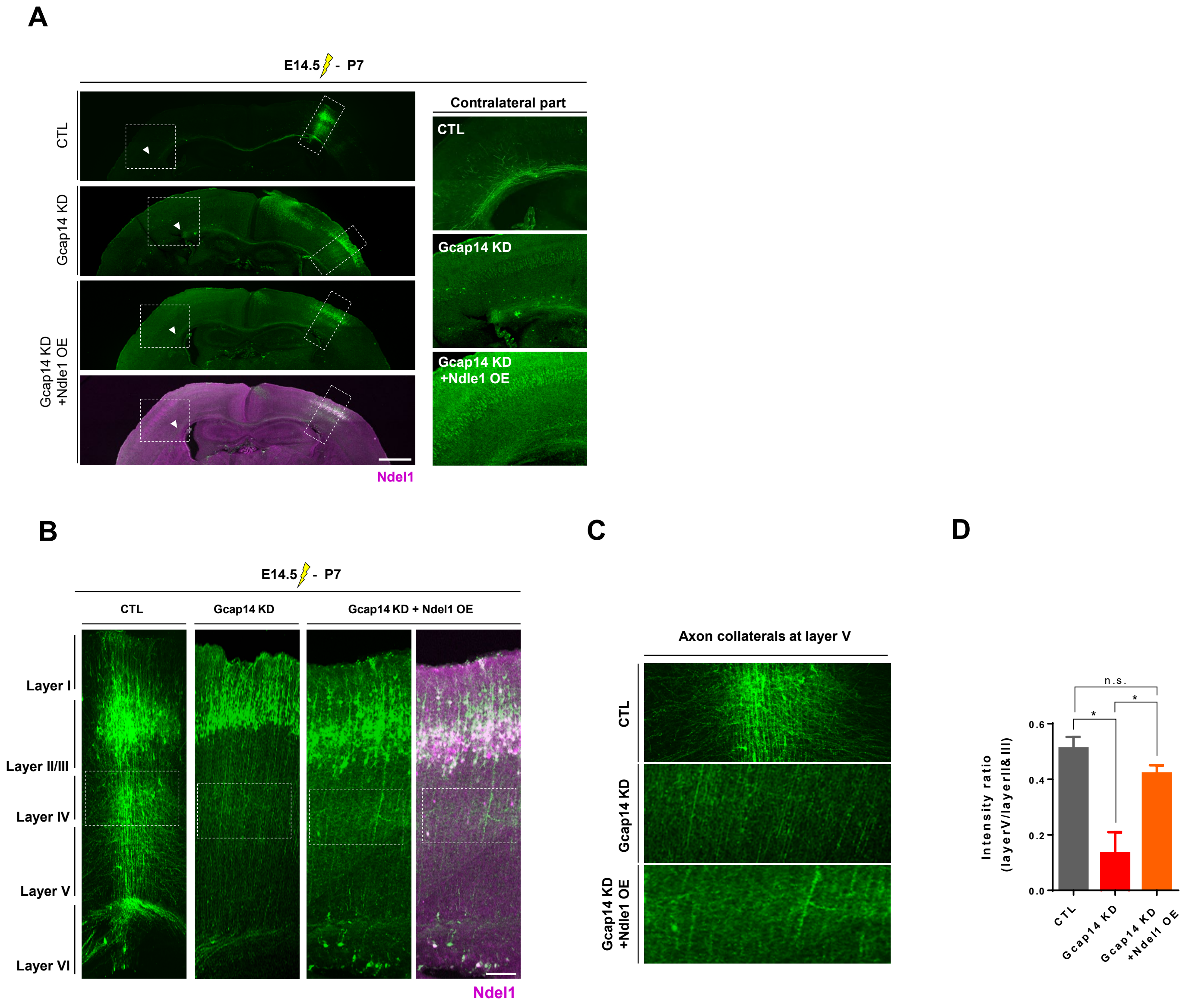

Fig S9.

The effects of Gcap14-Ndel1 complex on axon development, including ipsilateral branching and axon projection. (A) Representative image of brain of mouse embryos that were electroporated in utero with scrambled control and Gcap14 shRNA at E14.5 and analyzed at P7 (control, n=3 brains; Gcap14 shRNA, n=3 brains). Axons of transfected GFP-positive neurons (green) cross the midline, and the axonal branch was detected at the contralateral part of the control cortex. The scale bars represent 500μm. (B-C) Representative image of cortex and layer V. The scale bars represent 100μm. (D) Quantification of the extent of ipsilateral branching at the level of layer V neurons. All results are presented as mean ± SEM. *p<0.05, **p<0.01, ***p<0.001; NS, not significant by one-way ANOVA.
